# Clinical Characteristics and Outcomes in the Very Elderly Patients Hospitalized for Acute Heart Failure: Importance of Pharmacologic Guideline Adherence

**DOI:** 10.1038/s41598-018-32684-9

**Published:** 2018-09-24

**Authors:** Shih-Hsien Sung, Ta-Jung Wang, Hao-Min Cheng, Wen-Chung Yu, Chao-Yu Guo, Chern-En Chiang, Chen-Huan Chen

**Affiliations:** 1 Department of Medicine, Taipei Veterans General Hospital, Taipei, Taiwan; 2 Department of Medical Education, Taipei Veterans General Hospital, Taipei, Taiwan; 3 General Clinical Research Center, Taipei Veterans General Hospital, Taipei, Taiwan; 4 Cardiovascular Research Center, National Yang-Ming University, Taipei, Taiwan; 5 Department of Medicine, National Yang-Ming University, Taipei, Taiwan; 6 Institute of Public Health, National Yang-Ming University, Taipei, Taiwan; 7 Department of Medicine, Taipei Medical University Shuang Ho Hospital, Taipei, Taiwan; 8 Department of Internal Medicine, Taipei Medical University, Taipei, Taiwan

## Abstract

The prognostic factors and pharmacological effects of the very elderly patients (aged ≥80 years) with acute heart failure (AHF) remain unclear. The study, therefore, investigated the prognostic impacts of the guideline-recommended pharmacological therapy in these patients. A cohort of 1297 very elderly patients [85.1 ± 4.0 years, 69.7% male, 32.6% heart failure with reduced left ventricular ejection fraction (LVEF), HFrEF], hospitalized for AHF, was studied. The percentage of the recommended prescription for HFrEF at discharge, including renin-angiotensin system inhibitors, β-blockers, and mineralocorticoid receptor antagonists, was calculated as guideline adherence indicator (GAI). Among the 1233 survivors at discharge, 495 subjects (40.1%) died during a mean follow-up of 27.1 ± 23.9 months. Mean GAIs in HFrEF and HFpEF were 70.6 ± 34.9% and 64.1 ± 35.9%, respectively. A higher GAI was associated with less overall mortality [hazard ratio and 95% confidence interval per-1SD: 0.781, 0.655–0.930] and cardiovascular death (0.718, 0.558–0.925), independent of age, gender, diabetes, hypertension, mean blood pressure, LVEF, eGFR, sodium, and NT-proBNP. A GAI of 100% was associated with a better survival in both HFrEF and HFpEF. A prescription of the three recommended medications for HFrEF to the very elderly AHF patients was associated with a better survival after discharge.

## Introduction

Age is a major risk factor for heart failure (HF), and HF related outcomes, including hospitalization and death^1^. The incidence and prevalence of HF increase sharply with age and survival is dismal following the development of HF, especially in the elderly^1,2^. Due to a better management of chronic and acute HF patients, the survival after a diagnosis of HF has been improved over the past 30 years^1^. The age-standardized death rate has declined by 40% and the mean age at death from HF has risen from 80.0 to 82.7 years in seven European countries during two decades^3^. Despite the improvements, the five-year observed survival was about 26–52% for HF and was worse than that of many cancers and HF continues to be responsible for a tremendous burden on health care systems^1,4^.

Although the oldest old subjects (≥80 years) have the highest incidence, prevalence, and mortality of HF, the characteristics, management, and outcomes of the very elderly with HF have not been well described^5^, due to insufficient samples in most epidemiological surveys or registries^6–10^. The clinical picture of the octogenarian HF may differ substantially from that of the less old HF patients, because the progressive ventriculoarterial aging lowers the threshold for the development of HF^11^. The better survival of women and those with heart failure and preserved ejection fraction (HFpEF) contributes to the higher prevalence of HFpEF in the elderly women^12^.

More importantly, few land-mark HF trials have included very elderly patients and thus yielded limited evidence of pharmacological therapies in the octogenarian HF patients^11^. Furthermore, compared with younger patients, the elder HF patients often have problems with multiple comorbidities, and underuse and underdosage of the recommended drugs^5,13^, leading to suboptimal clinical outcomes^4,14,15^. International guidelines are not frequently implemented in this population, neither^16,17^. Whether the guideline-recommended treatments improve the clinical outcomes in the very elderly patients with HFrEF remains to be elucidated, especially when adverse drug events prevail among the very elderly^18^. So far, no treatment has been shown to improve outcomes in patients with HFpEF^17^. It is also unknown whether treatments recommended for HFrEF, including renin-angiotensin system (RAS) inhibitors, β-blockers, and mineralocorticoid receptor antagonists (MRAs), are tolerable for the oldest old HFpEF patients.

In the present study, we therefore investigated the prognostic impact of the guideline-recommended pharmacological therapy for HFrEF in the very elderly acute heart failure (AHF) patients, aged ≥80 years with HFrEF, as well as HFpEF.

## Methods

A total of 1297 patients aged over 80 years who were hospitalized primarily for AHF at Taipei Veterans General Hospital during the period from October 1, 2003, to December 31, 2012, was identified from HARVEST registry^19^. Patients with acute coronary syndrome, severe infection, severe hepatic disease, or active malignancy were excluded. Data of the index hospitalization on patient demographics, biochemistry, echocardiographic characteristics, co-morbidities, and medications, which have been prospectively registered in a web-based electronic medical recording system, were retrieved. The institutional review committee of Taipei Veterans General Hospital approved the use of the registry data for research purposes. Given the nature of an administrative registry, informed consent was waived.

Renal function, levels of serum electrolytes and N-terminal pro-B type natriuretic peptide (NT-proBNP) were measured at the presentations to the hospital. Lipid profiles were checked after 8 hours fasting in the next morning. Estimated glomerular filtration rate (eGFR) was calculated by the modified glomerular filtration rate estimating equation for Chinese patients^20^. There were missing values for NT-proBNP because the commercialized kit for NT-proBNP (Roche Diagnostics, Basel, Switzerland) was available after 2009. Echocardiography was performed by experienced technicians and independently reviewed by the physicians during hospitalization. LVEF was derived from the 2D-guided M-mode echocardiography^21^, and E/e’ was calculated as the ratio of early ventricular filling flow velocity (E) to the septal mitral annulus tissue velocity (e’). HFrEF and HFpEF were defined by LVEF < 50% and LVEF ≥ 50%, respectively^17^.

### Pharmacologic therapy and guideline adherence indicator

Medications on discharge were recorded. RAS inhibitors referred to either angiotensin-converting enzyme inhibitors or angiotensin receptor blockers. According to HF guidelines^16,17^, all 3 classes of life-saving medications, namely, RAS inhibitors, β-blockers, and MRAs, should be prescribed to patients with HFrEF in the absence of contraindications. The contraindications are renal insufficiency (eGFR < 30 ml/min/1.73 m^2^) or hyperkalemia (K > 5.5 mEq/L) for RAS inhibitors and MRAs, and bronchial asthma or profound bradycardia for β-blockers. When a patient with HFrEF received all of the indicated HF medications, he or she was considered to be 100% adhering to the guidelines. A guideline adherence indicator (GAI) was therefore calculated by dividing the number of prescribed medications by the number of indicated medications in percentage^15,22^. A GAI of 100% is considered to be complete adherence to the guidelines. Although the 3 classes of life-saving medications were not recommended to treat patients with HFpEF, a GAI was calculated for every patient.

### Follow up

The primary endpoints of mortality were confirmed by linking the database to the National Death Registry. The National Death Registry database registers valid information according to the International Classification of Disease, Ninth Revision (ICD-9). The ICD-9 codes for cardiovascular death were 390–459^23^.

### Statistical methods

Continuous variables were expressed as mean ± standard deviation (SD) and comparisons between groups were conducted by the Student’s t-test. Categorical data were described by the absolute number and relative frequencies and compared by the chi-square (χ^2^) test. The prognostic impact of GAI was analyzed using Kaplan–Meier accumulated survival curves. Multivariable Cox proportional hazards models were used to evaluate the independent predictors of mortality. Because the distribution of NT-proBNP was skewed, log transformation was conducted prior to Cox regression analysis. Subgroup analyses, stratified by age of 85 years, gender, the presence of diabetes, hypertension, or CAD, and renal function were conducted for GAI = 100%. In addition, patients with either HFrEF or HFpEF were analyzed for the prognostic impacts of GAI = 100% and each class of the 3 drugs. All statistics were performed by using SPSS v.16.0 software (SPSS, Inc., Chicago, IL, USA). All the tests performed were two-sided, and a P value < 0.05 was considered statistically significant.

## Results

### Patient characteristics and outcomes

The baseline characteristics of the 1297 patients (85.1 ± 4.0 years, 69.7% of men) are displayed in Table 1. The most common comorbidities in the study population were hypertension (66.1%), atrial fibrillation (33.8%), diabetes mellitus (31.9%), and coronary artery disease (27.9%), respectively. Comparing to patients with HFrEF (32.6%), patients with HFpEF were slightly older, more likely to be women, and had higher systolic blood pressure, pulse pressure, and lower heart rate at their presentation to the hospital. Hypertension was more prevalent in HFpEF, whereas coronary artery disease was more prevalent in HFrEF. In addition, HFpEF had higher E/e’, lower sodium levels, and lower NT-proBNP, in comparison to HFrEF. HFpEF and HFrEF had similar mean arterial blood pressure, left atrial diameter, right ventricular systolic blood pressure, eGFR, and prevalence of diabetes mellitus and atrial fibrillation.

**Table 1:** Baseline clinical characteristics of the study population, stratified by left ventricular ejection fraction.

	Total n = 1297	HFrEF n = 423	HFpEF n = 874	P value
Age, (year)	85.1 ± 4.0	84.5 ± 3.8	85.4 ± 4.0	<0.001
Male, n (%)	904 (69.7)	331 (78.3)	573 (65.6)	<0.001
Vital signs at the first presentation				
SBP, mmHg	147 ± 32	141 ± 30	149 ± 33	<0.001
MAP, mmHg	102 ± 21	100 ± 21	102 ± 21	0.133
PP, mmHg	67 ± 25	61 ± 22	70 ± 26	<0.001
Heart rate, beats/minute	90 ± 25	96 ± 27	87 ± 24	<0.001
Comorbidity, n (%)				
Hypertension	857 (66.1)	253 (59.8)	604 (69.1)	0.001
Diabetes mellitus	414 (31.9)	133 (32.1)	281 (32.2)	0.797
Coronary artery disease	362 (27.9)	152 (35.9)	210 (24.0)	<0.001
Atrial fibrillation	438 (33.8)	150 (35.5)	288 (33.0)	0.370
Echocardiogram				
Left atrial diameter, cm	4.5 ± 0.9	4.5 ± 0.9	4.6 ± 0.9	0.271
LVEF, %	57.0 ± 20.5	34.7 ± 18.1	67.8 ± 10.4	<0.001
RVSP, mmHg	42.9 ± 15.7	44.9 ± 8.8	45.5 ± 15.5	0.151
E/e’	17.4 ± 7.6	16.5 ± 7.1	19.4 ± 8.3	<0.001
Laboratory data				
eGFR, ml/min/1.73 m^2^	52.6 ± 26.6	52.2 ± 22.8	52.9 ± 28.3	0.670
Hemoglobin, mg/dl	11.4 ± 2.0	11.9 ± 1.9	11.2 ± 2.0	<0.001
Sodium, mEq/L	138.7 ± 5.1	139.1 ± 4.7	138.5 ± 5.3	0.041
Potassium, mEq/L	4.1 ± 0.7	4.0 ± 0.6	4.1 ± 0.7	0.082
*NT-proBNP, ng/L (n = 599)	5392 ± 3.7	9325 ± 2.7	4072 ± 3.9	<0.001

*Geometric mean and standard deviation.

E/e’ = ratio of early ventricular filling flow velocity (E) to the septal mitral annulus tissue velocity (e’); eGFR = estimated glomerular filtration rate; HFpEF = heart failure with preserved ejection fraction; HFrEF = heart failure with reduced ejection fraction; LVEF = left ventricular ejection fraction; MAP = mean arterial blood pressure; NT-proBNP = N-terminal prohormone brain natriuretic peptide; PP = pulse pressure; RVSP = right ventricular systolic pressure; SBP = systolic blood pressure.

In total, 64 inpatient deaths were recorded (4.9%). Among the 1233 survivors, 495 post-discharge deaths were observed during a mean follow-up of 27.1 ± 23.9 months. Table 2 reveals the on-discharge major pharmacotherapy and GAI in the discharged survivors. Comparing to HFpEF, patients with HFrEF were more likely to be put on β-blockers, MRAs and Digoxin. The prescriptions of RAS inhibitors, diuretics and nitrate were similar between the two groups. Average GAI was higher in HFrEF than HFpEF, and 297 of the 398 HFrEF patients (74.6%) were fully adherent to the 3 guidelines-recommended medications (GAI = 100%). In contrast, 565 of the 835 HFpEF patients (67.7%) had a GAI of 100%.

**Table 2:** On-discharge pharmacotherapy and guideline adherence indictor of the index hospitalization survivors (n = 1233).

	Total n = 1233	HFrEF n = 398	HFpEF n = 835	P value
Guideline adherence indictor, %	66.2 ± 35.7	70.6 ± 34.9	64.1 ± 35.9	0.003
Medications, n (%)				
Beta-blockers	706 (57.3)	246 (61.8)	460 (55.1)	0.026
RAS inhibitors	1041 (84.4)	344 (86.4)	697 (83.5)	0.180
MRAs	696 (56.4)	261 (65.6)	435 (52.1)	<0.001
Diuretics	1077 (87.3)	354 (88.9)	723 (86.6)	0.244
Digoxin	398 (32.3)	161 (40.5)	237 (28.4)	<0.001
Nitrate	856 (69.4)	286 (71.9)	570 (68.3)	0.200

HFpEF = heart failure with preserved ejection fraction; HFrEF = heart failure with reduced ejection fraction; MRAs = Mineralocorticoid receptor antagonists; RAS inhibitors = renin-angiotensin system inhibitors, including angiotensin converting enzyme inhibitors and angiotensin II receptor antagonists.

### Predictors of mortality in acute heart failure

**Figure 1:**
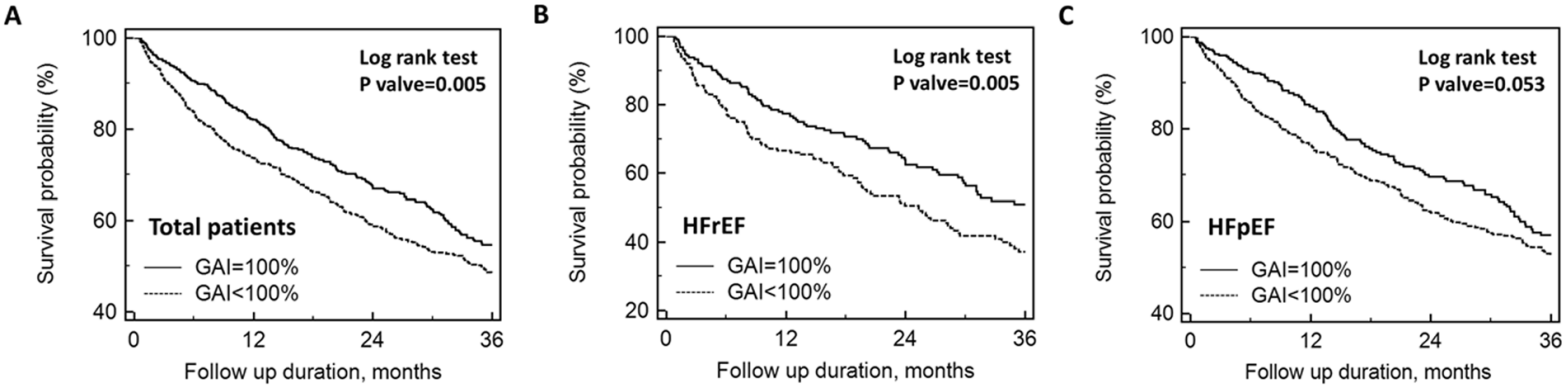
Kaplan–Meier survival curve. Kaplan–Meier survival curve analysis in total study population (**A**) and in patients with reduced left ventricular ejection fraction (HFrEF; **B**) or preserved left ventricular ejection fraction (HFpEF; **C**), stratified by the guideline adherence indicator (GAI).

In univariate analyses, older age, lower LVEF, eGFR, sodium, GAI, and higher NT-proBNP were significantly associated with post-discharge mortality in the whole study population (Supplementary Table S1). In the whole study population and the group of HFrEF, patients who had a GAI of 100% had a significantly better overall survival than those with a GAI < 100% over the entire follow-up period of 3 years (Fig. 1A, B). In patients with HFpEF, patients who had a GAI of 100% also had a significantly reduced overall mortality at the 1-year and 2-year follow-up (P = 0.003 and 0.013, respectively), but the difference became borderline significant (P = 0.053) at the end of the 3-year follow-up (Fig. 1C).

In multivariable analyses, a higher GAI was independently associated with lower mortality and cardiovascular death in the whole study population (hazard ratio and 95% confidence interval per-1SD: 0.840, 0.754–0.935 and 0.842, 0.712–0.996, respectively), after accounting for age, gender, diabetes mellitus, hypertension, mean blood pressure, left ventricular ejection fraction, estimated glomerular filtration rate and sodium (Table 3, Model 1). With a further adjustment for NT-proBNP, GAI remained significantly associated with total and cardiovascular mortality (0.781, 0.655–0.930 and 0.718, 0.558–0.925, respectively) (Table 3, Model 2).

**Table 3:** Predictors of post-discharge mortality in total study population (n = 1233).

guideline adherence indicator, 1sd = 35.68%	Crude hazard ratio		Model 1		Model 2	
	(95%CI)	P valve	(95%CI)	P valve	(95%CI)	P valve
Total mortality	0.836(0.768–0.911)	<0.001	**0**.**840(0**.**754–0**.**935)**	**0**.**001**	**0**.**781(0**.**655–0**.**930)**	**0**.**005**
CV mortality	0.827(0.726–0.944)	0.005	**0**.**842(0**.**712–0**.**996)**	**0**.**045**	**0**.**718(0**.**558–0**.**925)**	**0**.**010**

Model 1: adjusted for age, gender, diabetes mellitus, hypertension, mean blood pressure, left ventricular ejection fraction, estimated glomerular filtration rate and sodium.

Model 2: Model1 plus log transformation of N-terminal prohormone brain natriuretic peptide.

**Figure 2:**
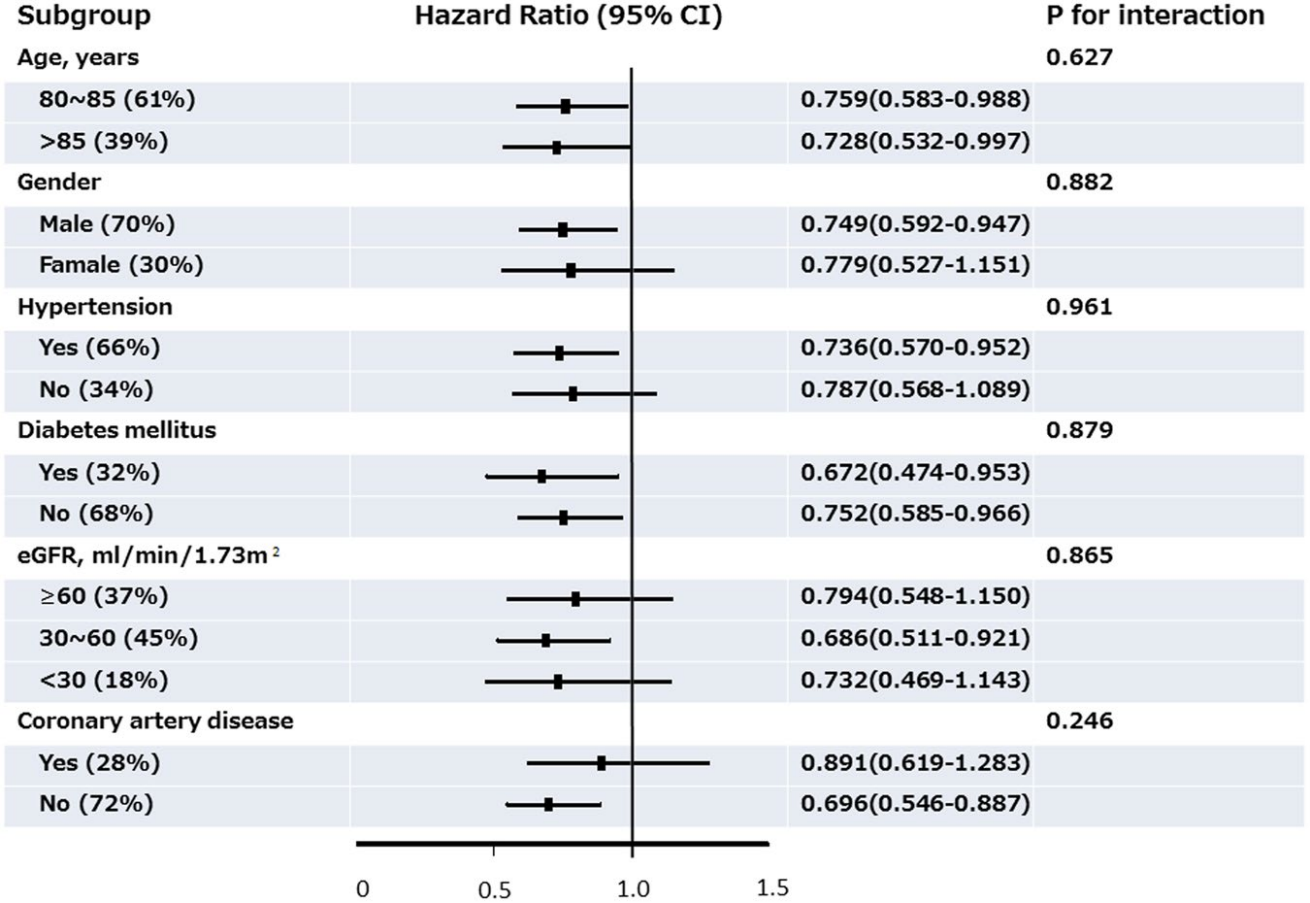
Forest plot for subgroup analysis. Hazard ratios (HRs) and 95% confidence intervals (CIs) for post-discharge mortality of a guideline adherence indicator of 100% versus <100% in subgroup analyses, after accounting for age, gender, left ventricular ejection fraction, and estimated glomerular filtration rate.

The subgroup analyses demonstrated that a GAI of 100% was consistently associated with a similar reduction of total mortality across various subpopulations, stratified by age, gender, presence of diabetes, hypertension, or coronary artery disease, and renal function, independent of age, gender, LVEF, and eGFR (Fig. 2).

### Pharmacotherapy and mortality in HFrEF and HFpEF

**Figure 3:**
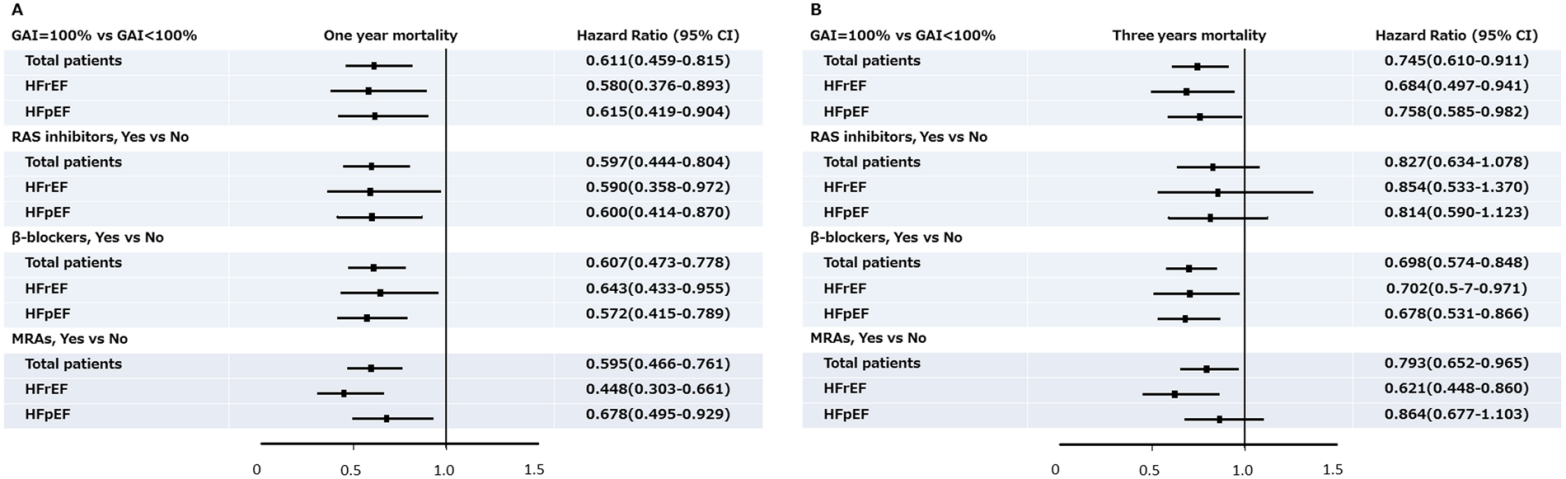
Hazard ratio of 1-year and 3-year total mortality. Hazard ratios and 95% confidence intervals (CI) for post-discharge 1-year (**A**) and 3-year (**B**) total mortality of guideline adherence indicator (GAI) = 100%, and the prescriptions of renin-angiotensin system (RAS) inhibitors, β-blockers, and mineralocorticoid receptor antagonists (MRAs), after accounting for age, gender, left ventricular ejection fraction, and estimated glomerular filtration rate. HFpEF = heart failure with preserved ejection fraction; HFrEF = heart failure with reduced ejection fraction.

With adjustments for age, gender, LVEF and eGFR, the on-discharge individual prescriptions of RAS inhibitors, β-blockers, and MRAs were significantly associated with a reduction of 1-year overall mortality in the total study population by 40.3%, 39.3%, and 40.5%, respectively, and also in patients with HFrEF (by 41%, 35.7%, and 55.2%, correspondingly) and HFpEF (by 40%, 42.8%, and 32.2%, correspondingly) (Fig. 3A). However, only β-blockers and MRAs but not RAS inhibitors were independently associated with a reduction of 3-year overall mortality in the total study population and in HFrEF (Fig. 3B). In HFpEF, only the prescription of β-blockers was independently associated with a better 3-year overall survival. In contrast, a GAI of 100% was consistently associated with a significantly lower 1-year and 3-year overall mortality in the whole study population, and also in the groups of HFrEF and HFpEF, independent of age, gender, LVEF and eGFR (Fig. 3A,B).

## Discussions

In this large very elderly cohort of patients hospitalized due to AHF, we found that HFpEF was more prevalent than HFrEF, but HFpEF and HFrEF shared similar clinical characteristics, comorbidities, echocardiographic findings, laboratory data, and even on-discharge medications, with some significant but small absolute differences. The on-discharge prescription of the 3 guidelines-recommended medications for HFrEF, was significantly and independently associated with post-discharge mortality in the very elderly AHF patients, with either HFrEF or HFpEF. In particular, the study results suggest that RAS inhibitors, β-blockers and MRAs may offer survival benefits at one year after discharge, and β-blockers may have prolonged survival benefits in both HFrEF and HFpEF. Thus, the results may encourage the guideline adherent pharmacological therapies in the very elderly HF patients to improve survival.

### Prognosis of the very elderly with acute heart failure

It has been noticed that the survival of the octogenarians with AHF was dismal, compared with patients of age <80 years^5^. The European heart failure surveys have demonstrated the octogenarians were used to have multiple co-morbidities, preserved LVEF, and higher mortality at both 12-week and 1-year follow-up duration^5^. Comparing with the 1-year mortality rate in European heart failure survey II of 28.4%, the post-discharge mortality rate in this study were 21.4% at 1-year, 36.7% at 2-year and 40.1% at 3-year follow-up. In addition to co-morbidities and medications, Komajda *et al*. suggested age and renal function were the independent baseline characteristics related to mortality^5^. In the present study, we further showed that LVEF, serum sodium and NT-proBNP levels, as well as age and eGFR, were independently related to long-term mortality. Although it has been suggested patients with either HFrEF or HFpEF shared similar risks of mortality^24^, the growing evidence may support the findings that LVEF was related to clinical outcomes^25^. Hyponatremia has been correlated with the prognosis of patients hospitalized for acute HF that a lower on-admission serum sodium level was related with a worse outcome^26,27^. While Barsheshet *et al*. have shown the prognostic discrepancies of hyponatremia in patients of ≤75 years or >75 years^28^, the present study expands that hyponatremia remains predictive of mortality in this very elderly population with acute HF.

### Guideline adherent prescriptions and mortality

Based on the recommendations from clinical trials^16,17^, pharmacological therapies including RAS inhibitors, β-blockers, and MRAs may attenuate the clinical risks of mortality and morbidity in patients with HFrEF^15,29^. While the majority of the clinical trials have excluded octogenarians, the present study suggested the guideline-adherent medications may save lives in the very elderly population with HFrEF.

Although RAS inhibitors, including ACE inhibitors and ARBs, have been beneficial to all stages of HFrEF^16,17^, Flather *et al*. demonstrated an attenuated effect of ACE inhibitors on mortality and morbidity with increasing age in a meta-analysis of 12763 chronic HF subjects with reduced LVEF^30^. In a mean follow-up duration of 35 months, ACE inhibitors were not related to death, hospitalization for HF, or myocardial infarction in patients aged >75 years^30^. In contrast, Masoudi *et al*. showed the 1-year survival benefit of ACE inhibitors persisted across all age subpopulations of acute HF, including subjects over 85 years^31^. In the present study, the prescription of RAS inhibitors was independently associated with a lower 1-year but not 3-year mortality in the very elderly patients, which may echo that the survival benefit of RAS inhibitors may attenuate with a longer follow-up duration^30^.

In a meta-analysis of 12729 subjects with chronic HF, Dulin *et al*. proposed that the elderly may get equal benefits from β-blockers, comparing to the non-elderly patients^32^. Nebivolol was associated with a 14% reduction of primary endpoints in the elderly HF patients of >70 years^33^. In a propensity-matched cohort of acute HF patients, the use of β-blockers was associated with lower 30-day and 4-year post-discharge mortality in the elderly of Medicare beneficiaries^34^. The present study further extends that the on-discharge prescription of β-blockers was independently associated with better 1-year and 3-year survival in the very elderly patients with HFrEF.

The survival advantage of MRAs in HFrEF has been documented in RALES and EPHESUS trial^35,36^, and the subgroup analyses suggested that the elderly (defined by ≥65, ≥67, or ≥75 years) had comparative benefits with MRAs. The present study furthers our understandings that MRAs may prolong survival even in the very elderly HFrEF patients.

### Pharmacological therapy for HFpEF

RAS inhibitors have not been proven to improve survival in HFpEF^1^. On the other hand, the PEP-CHF trial, composed of patients ≥70 years with HFpEF, demonstrated modest clinical benefits of ACE inhibitors at 1 year^37^. In a cohort of 438 Chinese HFpEF patients (mean age 64.7 ± 9.6 years), the prescription of angiotensin-converting enzyme inhibitors was associated with a significant decrease in overall mortality during a long-term follow-up^38^. The present study also supports that the prescription of RAS inhibitors may improve the 1-year survival of the very elderly HFpEF patients.

The effectiveness of β-blockers in the management of HFpEF has not been established. Two large observational trials of 11326 and 11959 elderly acute HF patients (mean age 73.9 and 78.4 years in Kaiser Permanente of Northern California and Medicare beneficiaries of north Carolina), respectively, proposed that the use of β-blockers was associated with favorable outcomes in a composite population of HFpEF and HFrEF^39,40^. A meta-analysis comprising 12 studies and 21206 subjects with HFpEF demonstrated that treatment with β-blockers was related to a significant reduction of mortality, both in patients <65 and ≥65 years^41^. Although conflict results exist^42^, the present study also showed a survival benefit of β-blockers in the very elderly patients with HFpEF up to 3 years follow-up. The result may encourage a therapeutic trial of β-blockers in the management of HFpEF in octogenarians.

While MRAs may significantly improve left ventricular diastolic function and serum markers of cardiac fibrosis in patients with HFpEF^43,44^, the prognostic impact of MRAs in HFpEF wasn’s encouraging in the TOPCAT trial^45^. Although spironolactone did not reduce mortality, hospitalization for HF was significantly lower in the spironolactone group^45^. Furthermore, Patel *et al*. demonstrated a real-world data that spironolactone might be associated with a favorable effect in HFpEF patients aged ≥80 years^46^. Similarly, the present study suggests that the prescription of MRAs may be beneficial in the very elderly HFpEF patients.

There were 58 patients with severe valvular heart disease in this study, including severe AS, severe AR, and severe MR, while surgery or transcatheter therapy might affect the prognoses. However, GAI was independently related to the long-term mortality in patients without severe valvular heart disease (0.841, 0.745–0.949), after accounting for age, gender, diabetes mellitus, hypertension, mean blood pressure, LVEF, eGFR, and sodium.

### Study Limitations

There were several limitations in this study. First, given the nature of an observational study of a single-center registry, there was a selection bias arising from unobserved variables. However, we have adjusted all available confounders to show the independent prognostic value of each potential predictor. The prognostic effects of RAS inhibitors, β-blockers and MRAs have been adjusted with age, gender, LVEF and eGFR that could influence the decisions of pharmacotherapy. Second, NT-proBNP was only available in 45.33% of the discharged survivors. In this subset of 559 patients with available NT-proBNP data, there were 175 deaths. We therefore still had sufficient power to demonstrate the prognostic value of GAI, independent of NT-proBNP. Third, the available outcomes were 6 years out of date. Therefore, there might be immortal time bias, and we would not be able to discuss the clinical impacts of the recently developed drugs for HF. The study was lack of non-fatal end-points, such as re-hospitalization for HF and quality of life. Given HF re-admission is a major prognostic factor, further studies are warranted to evaluate the associations of GAI with repeated hospitalization. Fourth, the bi-plane Simpson’s method was endorsed to the registry for the measure of LVEF in late 2010, and the data was only available in 377 patients. However, a Kappa value of 0.612 indicated substantial agreement on defining HFrEF by Simpson’s-derived EF of <40% or M-mode derived EF of <50%. A sensitivity analysis was conducted in 238 subjects with M-mode derived EF of <40% to show GAI was also associated with long-term mortality (0.808, 0.670–0.974). Furthermore, GAI is a pretty raw index of therapeutic adherence rather than the true medicine compliance. Since the follow-up data of biochemistry, vital signs, and the drug dose were not available, we were not able to calculate a better index according to whether or not the dose of each specific drugs was adequately titrated. Other well-known prognostic factors, including serum albumin, total bilirubin, and body mass index were not included in this analysis, neither. Further works are indicated to address the benefits of GAI on morbidities and mortalities, in the very elderly patients with acute HF.

## Conclusions

Guidelines-adherent prescriptions for HFrEF may prolong survival in the very elderly acute HF patients. Very elderly patients with acute HFpEF may also benefit from the RAS inhibitors, β-blockers, and MRAs. The study results may encourage physicians to prescribe the guidelines-recommended life-saving medications to the very elderly patients with HF.

## Electronic supplementary material

Supplementary Table S1
