# Supplementary material for: Clinical Characteristics and Outcomes in the Very Elderly Patients Hospitalized for Acute Heart Failure: Importance of Pharmacologic Guideline Adherence
Source: Sci Rep. 2018 Sep 24;8:14270. doi: 10.1038/s41598-018-32684-9 (PMC6155282; doi:10.1038/s41598-018-32684-9)
Supplement: Supplementary file 1 — Supplementary Table S1 [file 41598_2018_32684_MOESM1_ESM.pdf]

**Clinical Characteristics and Outcomes in the Very Elderly Patients Hospitalized for Acute Heart Failure: Importance of Pharmacologic Guideline Adherence**

Shih-Hsien Sung[a,e,f], Ta-Jung Wang[g,h], Hao-Min Cheng[b,d,e,f], Wen-Chung Yu[a,d,e], Chao-Yu Guo[f], Chern-En Chiang[c,d,e], *Chen-Huan Chen[b,d,e,f]

[a]Department of Medicine, [b]Department of Medical Education, and [c]General Clinical Research Center, Taipei Veterans General Hospital, Taipei, Taiwan; [d]Cardiovascular Research Center, [e]Department of Medicine, and [f]Institute of Public Health, National Yang-Ming University, Taipei, Taiwan; [g]Department of Medicine, Taipei Medical University Shuang Ho Hospital, Taipei, Taiwan; [h]Department of Internal Medicine, Taipei Medical University, Taipei, Taiwan

*Chen-Huan Chen is a co-corresponding author

Running title: Guideline adherence and Octogenarian heart failure

Conflict of Interest Disclosure: none

Address for correspondence:

Shih-Hsien Sung, M.D., Ph.D.

Division of Cardiology, Taipei Veterans General Hospital

201 Sec. 2, Shih-Pai Road, Taipei, Taiwan.

Tel: 886-2-2877-1746

Fax: 886-2-28771-746

E-mail: mr.sungsh@gmail.com

Table S1. Univariate analysis for the predictors of post-discharge mortality (n=1233)

| Variable | HR (95% CI) | P valve |
|---|---|---|
| Age, 1sd=3.95 years | 1.140(1.047-1.240) | 0.002 |
| †Gender | 0.908(0.748-1.103) | 0.333 |
| MAP, 1sd=21mmHg | 0.951(0.861-1.051) | 0.325 |
| ‡Hypertension | 0.756(0.632-0.906) | 0.002 |
| ‡Diabetes | 1.098(0.911-1.324) | 0.326 |
| ‡Coronary artery disease | 0.930(0.762-1.135) | 0.477 |
| LVEF, 1sd=20.6% | 0.893(0.832-0.958) | 0.002 |
| eGFR, 1sd=26.2 ml/min/1.73m$^2$ | 0.897(0.806-0.997) | 0.045 |
| Sodium, 1sd=5.03 mEq/L | 0.905(0.828-0.990) | 0.029 |
| Log NT-proBNP, 1sd=1.3 pg/ml (n=559) | 1.450(1.240-1.696) | <0.001 |
| Guideline adherence indicator, 1sd=35.68 % | 0.836(0.768-0.911) | <0.001 |

† women versus men; ‡ yes versus no

eGFR= estimated glomerular filtration rate; LVEF= left ventricular ejection fraction;

MAP=mean arterial blood pressure; NT-proBNP= N-terminal pro-brain natriuretic peptide.
